# Supplementary material for: Tradeoffs explain scaling, sex differences, and seasonal oscillations in the remarkable weapons of snapping shrimp (Alpheus spp.)
Source: eLife. 2023 May 9;12:e84589. doi: 10.7554/eLife.84589 (PMC10234628; doi:10.7554/eLife.84589)
Supplement: Supplementary file 1. [file elife-84589-supp1.docx]

**Supplemental Materials**

Article: Tradeoffs and Benefits Explain Scaling, Sex Differences, and Seasonal Oscillations in the Remarkable Weapons of Snapping Shrimp (*Alpheus spp.*)

Jason P. Dinh, S.N. Patek

Biology Department, Duke University

***Supplemental Table 1:*** *Scaling slopes for log_10_(snapping claw length) and log_10_(abdomen length) as a function of log_10_(rostrum-to-telson length). All measurements were taken in millimeters. 95% confidence intervals for slopes are shown in brackets after the slope estimate.*

|  | *A. heterochaelis* males | *A. heterochaelis* females | *A. angulosus* males | *A. angulosus* females | *A. estuariensis* males | *A. estuariensis* females |
| --- | --- | --- | --- | --- | --- | --- |
| Snapping claw scaling slope | 1.193 [1.149 1.238] | 1.000 [0.967 1.033] | 1.489 [0.934 2.044] | 1.013 [0.709 1.308] | 1.207 [1.096 1.318] | 1.173 [1.029 1.316] |
| Abdomen scaling slope | 1.027 [1.013 1.042] | 1.015 [1.004 1.026] | 0.934 [0.837 1.031] | 1.064 [0.978 1.149] | 0.934 [0.876 0.992] | 1.064 [1.007 1.121] |

***Supplemental Table 2****: Model summary for abdomen-snapping claw tradeoffs in* Alpheus angulosus.

|  | |
| --- | --- |
|  | Dependent variable: |
|  |  |
|  | Abdomen Residual |
|  | |
| Snapping Claw Residual | -0.113^***^ |
|  | (0.026) |
|  |  |
| Constant | -0.000 |
|  | (0.002) |
|  |  |
|  | |
| Observations | 53 |
| R^2^ | 0.273 |
| Adjusted R^2^ | 0.259 |
| Residual Std. Error | 0.012 (df = 51) |
| F Statistic | 19.142^***^ (df = 1; 51) |
|  | |
| Note: | ^*^p^**^p^***^p<0.005 |

***Supplemental Table 3****: Model summary for abdomen-snapping claw tradeoffs in* Alpheus estuariensis*.*

|  | |
| --- | --- |
|  | Dependent variable: |
|  |  |
|  | Abdomen Residual |
|  | |
| Snapping Claw Residual | -0.142^*^ |
|  | (0.064) |
|  |  |
| Constant | 0.000 |
|  | (0.001) |
|  |  |
|  | |
| Observations | 45 |
| R^2^ | 0.103 |
| Adjusted R^2^ | 0.082 |
| Residual Std. Error | 0.007 (df = 43) |
| F Statistic | 4.924^*^ (df = 1; 43) |
|  | |
| Note: | ^*^p^**^p^***^p<0.005 |

***Supplemental Table 4****: Model summary for abdomen-snapping claw tradeoffs in* Alpheus heterochaelis*.*

|  | |
| --- | --- |
|  | Dependent variable: |
|  |  |
|  | Abdomen Residual |
|  | |
| Snapping Claw Residual | -0.123^***^ |
|  | (0.012) |
|  |  |
| Constant | -0.000 |
|  | (0.0004) |
|  |  |
|  | |
| Observations | 677 |
| R^2^ | 0.139 |
| Adjusted R^2^ | 0.138 |
| Residual Std. Error | 0.010 (df = 675) |
| F Statistic | 109.257^***^ (df = 1; 675) |
|  | |
| Note: | ^*^p<0.05; ^**^p<0.01; ^***^p<0.005 |

***Supplemental Table 5:*** *Model summary for size-dependent tradeoff between abdomen residuals and snapping claw residuals for* Alpheus heterochaelis *males. Positive interaction term indicates that as carapace length increases, the tradeoff between snapping claw residuals and abdomen residuals decreases.*

|  | |
| --- | --- |
|  | *Dependent variable:* |
|  |  |
|  | Abdomen Residual |
|  | |
| Snapping Claw Residual | -0.387^***^ |
|  | (0.085) |
|  |  |
| Carapace Length | -0.001^***^ |
|  | (0.0003) |
|  |  |
| Interaction | 0.025^***^ |
|  | (0.008) |
|  |  |
| Constant | 0.011^***^ |
|  | (0.003) |
|  |  |
|  | |
| Observations | 329 |
| R^2^ | 0.234 |
| Adjusted R^2^ | 0.227 |
| Residual Std. Error | 0.009 (df = 325) |
| F Statistic | 33.166^***^ (df = 3; 325) |
|  | |
| *Note:* | ^*^p<0.05; ^**^p<0.01; ^***^p<0.005 |

***Supplemental Table 6:*** *Model summary showing no size-dependent tradeoff between abdomen residuals and snapping claw residuals for* Alpheus heterochaelis *females. Interaction p > 0.05.*

|  | |
| --- | --- |
|  | Dependent variable: |
|  |  |
|  | Abdomen Residual |
|  | |
| Snapping Claw Residual | -0.119^*^ |
|  | (0.060) |
|  |  |
| Carapace Length | -0.001^***^ |
|  | (0.0003) |
|  |  |
| Interaction | 0.001 |
|  | (0.006) |
|  |  |
| Constant | 0.009^***^ |
|  | (0.003) |
|  |  |
|  | |
| Observations | 348 |
| R^2^ | 0.154 |
| Adjusted R^2^ | 0.147 |
| Residual Std. Error | 0.010 (df = 344) |
| F Statistic | 20.920^***^ (df = 3; 344) |
|  | |
| Note: | ^*^p<0.05; ^**^p<0.01; ^***^p<0.005 |
|  |  |

***Supplemental Table 7:*** *Model summary showing that snapping claw residuals did not predict female maximal sound pressure level.*

|  | |
| --- | --- |
|  | Dependent variable: |
|  |  |
|  | Max Sound Pressure Level (dB re 1 uPa) |
|  | |
| log_10_(Claw Mass) | 24.495^***^ |
|  | (3.717) |
|  |  |
| Snapping Claw Residual | 52.646 |
|  | (134.394) |
|  |  |
| Interaction | 79.314 |
|  | (132.893) |
|  |  |
| Constant | 213.726^***^ |
|  | (3.647) |
|  |  |
|  | |
| Observations | 40 |
| R^2^ | 0.572 |
| Adjusted R^2^ | 0.537 |
| Residual Std. Error | 6.239 (df = 36) |
| F Statistic | 16.049^***^ (df = 3; 36) |
|  | |
| Note: | ^*^p<0.05; ^**^p<0.01; ^***^p<0.005 |

***Supplemental Table 8:*** *Model summary showing that snapping claw residuals did not predict female maximal bubble duration.*

|  | |
| --- | --- |
|  | Dependent variable: |
|  |  |
|  | Max Bubble Duration (sec) |
|  | |
| log_10_(Claw Mass) | 0.0004^***^ |
|  | (0.00003) |
|  |  |
| Snapping Claw Residual | 0.0004 |
|  | (0.001) |
|  |  |
| Interaction | 0.0004 |
|  | (0.001) |
|  |  |
| Constant | 0.001^***^ |
|  | (0.00003) |
|  |  |
|  | |
| Observations | 40 |
| R^2^ | 0.850 |
| Adjusted R^2^ | 0.837 |
| Residual Std. Error | 0.00004 (df = 36) |
| F Statistic | 67.783^***^ (df = 3; 36) |
|  | |
| Note: | ^*^p<0.05; ^**^p<0.01; ^***^p<0.005 |

***Supplemental Table 9:*** *Model summary showing that snapping claw residuals did not predict female maximal average angular velocity.*

|  | |
| --- | --- |
|  | Dependent variable: |
|  |  |
|  | Max Average Angular Velocity (rad/sec) |
|  | |
| log_10_ (Claw Mass) | -2,486.871^***^ |
|  | (181.963) |
|  |  |
| Snapping Claw Residual | 3,478.511 |
|  | (6,579.674) |
|  |  |
| Interaction | 5,234.176 |
|  | (6,506.190) |
|  |  |
| Constant | -63.338 |
|  | (178.534) |
|  |  |
|  | |
| Observations | 40 |
| R^2^ | 0.842 |
| Adjusted R^2^ | 0.828 |
| Residual Std. Error | 305.445 (df = 36) |
| F Statistic | 63.757^***^ (df = 3; 36) |
|  | |
| Note: | ^*^p<0.05; ^**^p<0.01; ^***^p<0.005 |

***Supplemental Table 10:*** *Model summary showing that snapping claw residuals did not predict male maximal sound pressure level.*

|  | |
| --- | --- |
|  | Dependent variable: |
|  |  |
|  | Max Sound Pressure Level (dB re 1 uPa) |
|  | |
| log_10_ (Claw Mass) | 26.502^***^ |
|  | (2.177) |
|  |  |
| Snapping Claw Residual | 56.663 |
|  | (85.788) |
|  |  |
| Interaction | -25.760 |
|  | (84.560) |
|  |  |
| Constant | 213.511^***^ |
|  | (1.977) |
|  |  |
|  | |
| Observations | 36 |
| R^2^ | 0.863 |
| Adjusted R^2^ | 0.851 |
| Residual Std. Error | 4.280 (df = 32) |
| F Statistic | 67.432^***^ (df = 3; 32) |
|  | |
| Note: | ^*^p<0.05; ^**^p<0.01; ^***^p<0.005 |

***Supplemental Table 11:*** *Model summary showing that snapping claw residuals did not predict male maximal bubble duration.*

|  | |
| --- | --- |
|  | Dependent variable: |
|  |  |
|  | Max Bubble Duration (sec) |
|  | |
| log_10_ (Claw Mass) | 0.001^***^ |
|  | (0.00003) |
|  |  |
| Snapping Claw Residual | 0.001 |
|  | (0.001) |
|  |  |
| Interaction | 0.002 |
|  | (0.001) |
|  |  |
| Constant | 0.001^***^ |
|  | (0.00003) |
|  |  |
|  | |
| Observations | 36 |
| R^2^ | 0.909 |
| Adjusted R^2^ | 0.901 |
| Residual Std. Error | 0.0001 (df = 32) |
| F Statistic | 107.068^***^ (df = 3; 32) |
|  | |
| Note: | ^*^p<0.05; ^**^p<0.01; ^***^p<0.005 |

***Supplemental Table 12:*** *Model summary showing that snapping claw residuals did not predict male maximal average angular velocity.*

|  | |
| --- | --- |
|  | Dependent variable: |
|  |  |
|  | Max Average Angular Velocity (rad/sec) |
|  | |
| log_10_ (Claw Mass) | -1,370.772^***^ |
|  | (200.123) |
|  |  |
| Snapping Claw Residual | 3,025.541 |
|  | (7,887.771) |
|  |  |
| Interaction | -2,657.354 |
|  | (7,774.900) |
|  |  |
| Constant | 859.746^***^ |
|  | (181.787) |
|  |  |
|  | |
| Observations | 36 |
| R^2^ | 0.604 |
| Adjusted R^2^ | 0.567 |
| Residual Std. Error | 393.525 (df = 32) |
| F Statistic | 16.269^***^ (df = 3; 32) |
|  | |
| Note: | ^*^p<0.05; ^**^p<0.01; ^***^p<0.005 |

***Supplemental Table 13:*** *Summary tables for models showing tradeoffs between snapping claw residuals and egg mass volume residuals.*

|  | |
| --- | --- |
|  | Dependent variable: |
|  |  |
|  | Egg Mass Volume Residual |
|  | |
| Snapping claw residual | -630.261^*^ |
|  | (250.982) |
|  |  |
| Constant | 2.238 |
|  | (4.247) |
|  |  |
|  | |
| Observations | 37 |
| R^2^ | 0.153 |
| Adjusted R^2^ | 0.128 |
| Residual Std. Error | 25.258 (df = 35) |
| F Statistic | 6.306^*^ (df = 1; 35) |
|  | |
| Note: | ^*^p<0.05; ^**^p<0.01; ^***^p<0.005 |

***Supplemental Table 13:*** *Summary tables for models showing tradeoffs between snapping claw residuals and egg count residuals.*

|  | |
| --- | --- |
|  | Dependent variable: |
|  |  |
|  | Egg Count Residual |
|  | |
| Snapping Claw Residual | -1,120.278^*^ |
|  | (546.016) |
|  |  |
| Constant | 3.979 |
|  | (9.239) |
|  |  |
|  | |
| Observations | 37 |
| R^2^ | 0.107 |
| Adjusted R^2^ | 0.082 |
| Residual Std. Error | 54.949 (df = 35) |
| F Statistic | 4.210^*^ (df = 1; 35) |
|  | |
| Note: | ^*^p<0.05; ^**^p<0.01; ^***^p<0.005 |

***Supplemental Table 14:*** *Summary tables for models showing tradeoffs between snapping claw residuals and average egg volume.*

|  | |
| --- | --- |
|  | Dependent variable: |
|  |  |
|  | Average Egg Volume (mm^3^) |
|  | |
| Snapping Claw Residual | -1.368^*^ |
|  | (0.630) |
|  |  |
| Constant | 0.461^***^ |
|  | (0.011) |
|  |  |
|  | |
| Observations | 37 |
| R^2^ | 0.119 |
| Adjusted R^2^ | 0.094 |
| Residual Std. Error | 0.063 (df = 35) |
| F Statistic | 4.715^*^ (df = 1; 35) |
|  | |
| Note: | ^*^p<0.05; ^**^p<0.01; ^***^p<0.005 |

***Supplemental Table 15:*** *Interaction between carapace length and snapping claw residuals show that average egg volume/weapon tradeoffs are strongest for small individuals.*

|  | |
| --- | --- |
|  | Dependent variable: |
|  |  |
|  | Average Egg Volume (mm^3^) |
|  | |
| Snapping Claw Residual | -15.852^*^ |
|  | (6.306) |
|  |  |
| Carapace Length (mm) | -0.0003 |
|  | (0.010) |
|  |  |
| Interaction | 1.241^*^ |
|  | (0.538) |
|  |  |
| Constant | 0.456^***^ |
|  | (0.116) |
|  |  |
|  | |
| Observations | 37 |
| R^2^ | 0.241 |
| Adjusted R^2^ | 0.172 |
| Residual Std. Error | 0.061 (df = 33) |
| F Statistic | 3.496^*^ (df = 3; 33) |
|  | |
| Note: | ^*^p<0.05; ^**^p<0.01; ^***^p<0.005 |

***Supplemental Table 16:*** *ANOVA table testing if snapping claw residuals are predicted by sex, paired status, and their interaction. Both paired status and its interaction with sex were significant predictors. The directionality and strength of the coefficients suggests that the effect is strong in males and negligible in females.*

|  | **Df** | **Sum Sq** | **Mean Sq** | **F value** | **Pr(>F)** |
| --- | --- | --- | --- | --- | --- |
| male | 1 | 0.00111 | 0011111 | 1.460 | 0.227209 |
| paired | 1 | 0.00615 | 0.0061506 | 8.0926 | 0.004634** |
| male:paired | 1 | 0.00336 | 0.003366 | 4.4155 | 0.036132* |
| Residuals | 486 | 0.3711264 | 0.0007636 | NA | NA |

^*^p<0.05; ^**^p<0.01; ^***^p<0.005

***Supplemental Table 17:*** *Model summary showing that male pairing success is a function of snapping claw residual but not carapace length.*

|  | |
| --- | --- |
|  | Dependent variable: |
|  |  |
|  | Paired |
|  | |
| Snapping Claw Residual | 16.876^***^ |
|  | (5.770) |
|  |  |
| Carapace Length (mm) | 0.137 |
|  | (0.084) |
|  |  |
| Constant | -1.459 |
|  | (0.859) |
|  |  |
|  | |
| Observations | 233 |
| Log Likelihood | -153.156 |
| Akaike Inf. Crit. | 312.311 |
|  | |
| Note: | ^*^p<0.05; ^**^p<0.01; ^***^p<0.005 |

***Supplemental Table 18:*** *Model summary showing that female pairing success is a function of carapace length but not snapping claw residual.*

|  | |
| --- | --- |
|  | Dependent variable: |
|  |  |
|  | Paired |
|  | |
| Snapping Claw Residual | 3.384 |
|  | (4.872) |
|  |  |
| Carapace Length (mm) | 0.284^***^ |
|  | (0.069) |
|  |  |
| Constant | -3.188^***^ |
|  | (0.732) |
|  |  |
|  | |
| Observations | 253 |
| Log Likelihood | -163.753 |
| Akaike Inf. Crit. | 333.505 |
|  | |
| Note: | ^*^p<0.05; ^**^p<0.01; ^***^p<0.005 |

***Supplemental Table 19:*** *Snapping claw residual did not predict the relative rostrum-to-telson length of their partner in females.*

|  | |
| --- | --- |
|  | Dependent variable: |
|  |  |
|  | Partner's Relative Rostrum-to-Telson Length |
|  | |
| Snapping Claw Residual | 0.659 |
|  | (0.353) |
|  |  |
| Constant | -0.057^***^ |
|  | (0.008) |
|  |  |
|  | |
| Observations | 111 |
| R^2^ | 0.031 |
| Adjusted R^2^ | 0.022 |
| Residual Std. Error | 0.088 (df = 109) |
| F Statistic | 3.477 (df = 1; 109) |
|  | |
| Note: | ^*^p<0.05; ^**^p<0.01; ^***^p<0.005 |

***Supplemental Table 20:*** *Snapping claw residuals predicted the relative rostrum-to-telson length of their partner in males.*

|  | |
| --- | --- |
|  | Dependent variable: |
|  |  |
|  | Partner's Relative Rostrum-to-Telson Length |
|  | |
| Snapping Claw Residual | 0.878^***^ |
|  | (0.304) |
|  |  |
| Constant | 0.039^***^ |
|  | (0.008) |
|  |  |
|  | |
| Observations | 111 |
| R^2^ | 0.071 |
| Adjusted R^2^ | 0.063 |
| Residual Std. Error | 0.079 (df = 109) |
| F Statistic | 8.344^***^ (df = 1; 109) |
|  | |
| Note: | ^*^p<0.05; ^**^p<0.01; ^***^p<0.005 |

***Supplemental Table 21:*** *Seasonal morphological shift t-tests. Each t-test represents a row in the table, and the response variable for that t-test is shown in the left-most column.*

|  | **Difference between groups** | **Nonbreeding Snapping Claw Residuals** | **Breeding Snapping Claw Residuals** | **t** | **p.value** | **df** |
| --- | --- | --- | --- | --- | --- | --- |
| **Female Snapping claw Residual** | -0.0031416 | -0.0026902 | 0.0004514 | -0.6415478 | 0.5232794 | 69.4235 |
| **Male Snapping claw Residual** | -0.0215942 | -0.0183781 | 0.0032162 | -5.520575 | 4e-07*** | 81.24774 |
| **Female Abdomen Residual** | 0.0026658 | 0.0022828 | -0.000383 | 1.886613 | 0.0628752 | 79.24422 |
| **Male Abdomen Residual** | 0.0041563 | 0.0035373 | -0.000619 | 2.832303 | 0.0059894** | 71.99196 |
|  |  |  |  |  |  |  |

^*^p<0.05; ^**^p<0.01; ^***^p<0.005

***Supplemental Table 22:*** *Model summary showing female seasonal shifts in allometry.*

|  | |
| --- | --- |
|  | Dependent variable: |
|  |  |
|  | log_10_(Snapping Claw Length (mm)) |
|  | |
| log_10_(Rostrum-to-Telson Length (mm)) | 1.163^***^ |
|  | (0.051) |
|  |  |
| Breeding | 0.262^***^ |
|  | (0.077) |
|  |  |
| Interaction | -0.183^***^ |
|  | (0.054) |
|  |  |
| Constant | -0.588^***^ |
|  | (0.072) |
|  |  |
|  | |
| Observations | 348 |
| R^2^ | 0.914 |
| Adjusted R^2^ | 0.913 |
| Residual Std. Error | 0.033 (df = 344) |
| F Statistic | 1,216.580^***^ (df = 3; 344) |
|  | |
| Note: | ^*^p<0.05; ^**^p<0.01; ^***^p<0.005 |

***Supplemental Table 23:*** *Model summary showing no male seasonal shifts in scaling slope.*

|  | |
| --- | --- |
|  | Dependent variable: |
|  |  |
|  | log_10_(Snapping Claw Length (mm)) |
|  | |
| log_10_(Rostrum-to-Telson Length (mm)) | 1.234^***^ |
|  | (0.056) |
|  |  |
| Breeding | 0.126 |
|  | (0.086) |
|  |  |
| Interaction | -0.073 |
|  | (0.061) |
|  |  |
| Constant | -0.640^***^ |
|  | (0.079) |
|  |  |
|  | |
| Observations | 329 |
| R^2^ | 0.905 |
| Adjusted R^2^ | 0.904 |
| Residual Std. Error | 0.031 (df = 325) |
| F Statistic | 1,029.595^***^ (df = 3; 325) |
|  | |
| Note: | ^*^p<0.05; ^**^p<0.01; ^***^p<0.005 |

***Supplemental Table 24:*** *Model summary showing male seasonal upward shift snapping claw size.*

|  | |
| --- | --- |
|  | Dependent variable: |
|  |  |
|  | log_10_(Snapping Claw Length (mm)) |
|  | |
| log_10_(Rostrum-to-Telson Length (mm)) | 1.172^***^ |
|  | (0.022) |
|  |  |
| Breeding | 0.023^***^ |
|  | (0.005) |
|  |  |
| Constant | -0.553^***^ |
|  | (0.031) |
|  |  |
|  | |
| Observations | 329 |
| R^2^ | 0.904 |
| Adjusted R^2^ | 0.904 |
| Residual Std. Error | 0.031 (df = 326) |
| F Statistic | 1,541.657^***^ (df = 2; 326) |
|  | |
| Note: | ^*^p<0.05; ^**^p<0.01; ^***^p<0.005 |

*
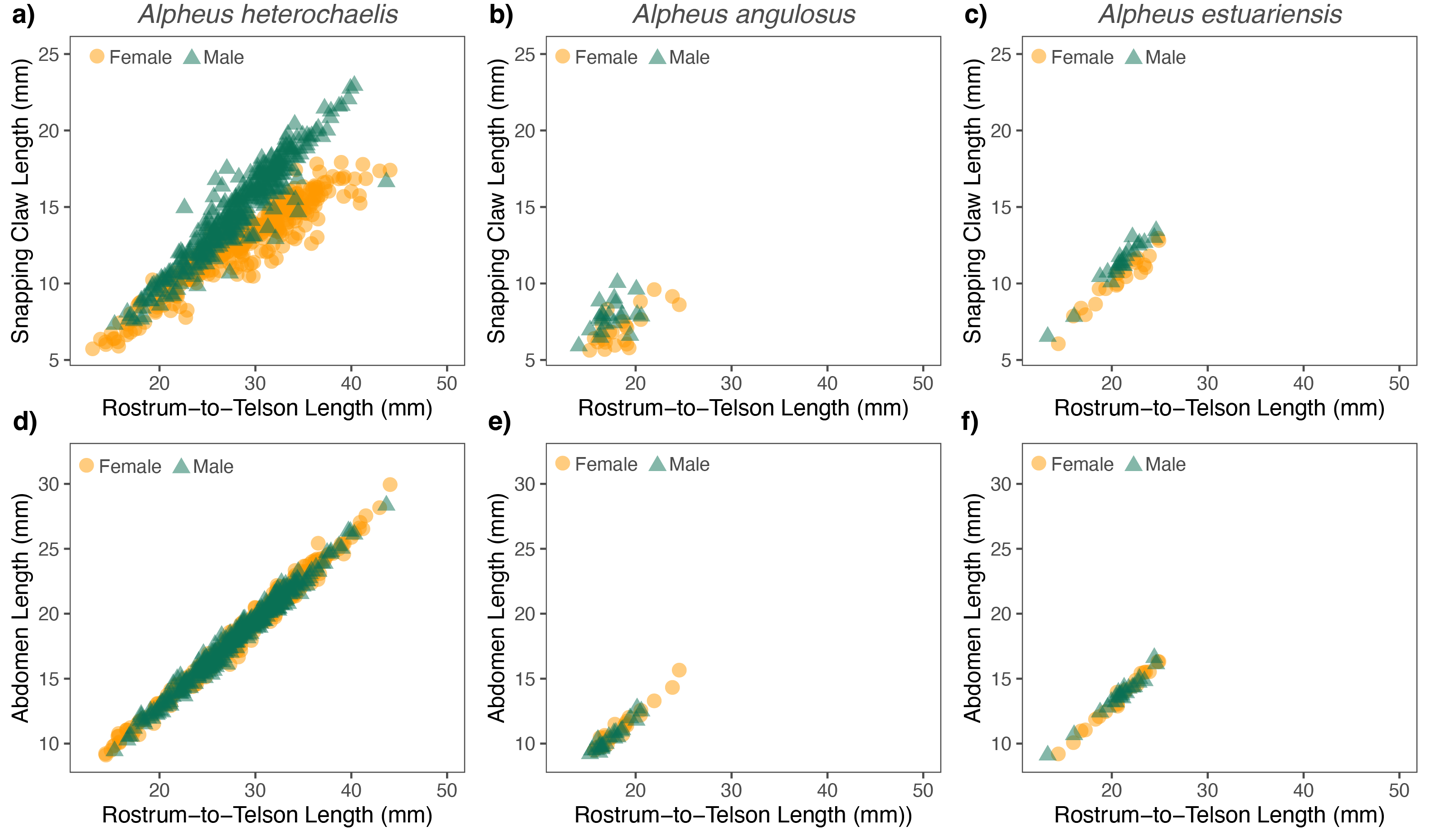
*

***Supplemental Figure 1:*** *Scaling relationships for snapping claw length and abdomen length shown in linear scaling.*


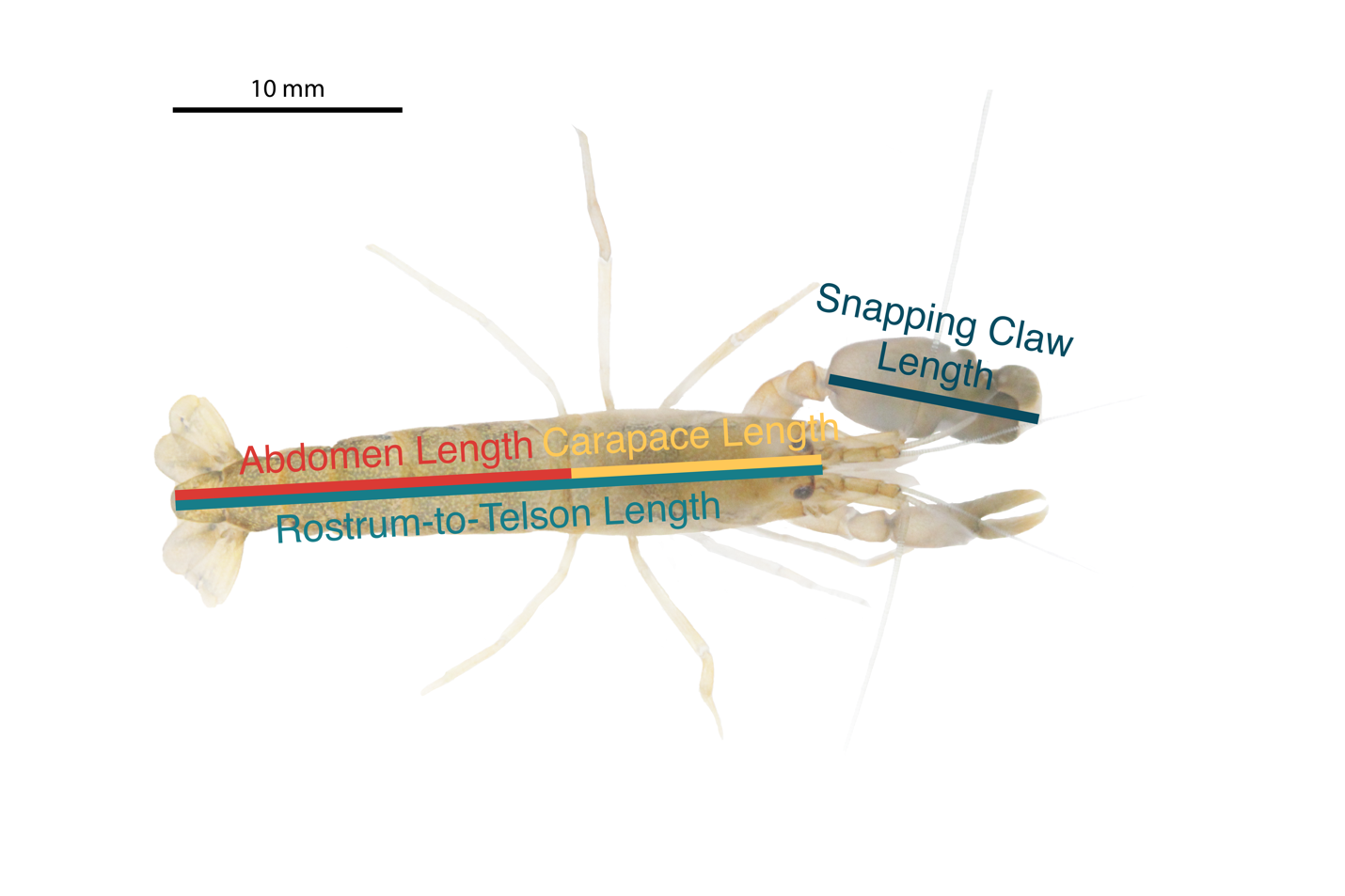
***Supplemental Figure 2:*** *Morphological measurements used in this study. Example shown is an* Alpheus angulosus *female.*
